# Is the Surface Potential Integral of a Dipole in a Volume Conductor Always Zero? A Cloud Over the Average Reference of EEG and ERP

**DOI:** 10.1007/s10548-016-0543-x

**Published:** 2017-02-14

**Authors:** Dezhong Yao

**Affiliations:** Key Laboratory for NeuroInformation of Ministry of Education, Center for Information in Medicine, University of Electronic Science and Technology of China, Chengdu, 610054 China

**Keywords:** Average reference, Zero potential, Spherical head, Volume conductor model, Gold standard

## Abstract

Currently, average reference is one of the most widely adopted references in EEG and ERP studies. The theoretical assumption is the surface potential integral of a volume conductor being zero, thus the average of scalp potential recordings might be an approximation of the theoretically desired zero reference. However, such a zero integral assumption has been proved only for a spherical surface. In this short communication, three counter-examples are given to show that the potential integral over the surface of a dipole in a volume conductor may not be zero. It depends on the shape of the conductor and the orientation of the dipole. This fact on one side means that average reference is not a theoretical ‘gold standard’ reference, and on the other side reminds us that the practical accuracy of average reference is not only determined by the well-known electrode array density and its coverage but also intrinsically by the head shape. It means that reference selection still is a fundamental problem to be fixed in various EEG and ERP studies.

## Background

Since human EEG was reported in 1929, the reference issue has been a long-term debate as only the potential difference between two points can be measured (Dien 1988; Schiff 2005). It is indispensable to have a reference for scalp recording; however, there is no point on the scalp or body surface where the potential is zero or a constant. The ever-present experienced cephalic reference electrodes include the uni-mastoid or ear, linked mastoids or ears (LMR), the vertex, the tip of the nose, neck ring, etc. To reduce the effect of a non-zero actual reference on various studies, a few off-line re-reference techniques have been proposed in the past years, and among them, the average reference (AR) (Goldman 1950; Offner 1950) has often been advocated as the best available reference option (Nunez 2010), as Nunez and Srinivasan (2006) stated, “…when used with large numbers of electrodes…it often performs reasonably well…”. In a recent paper, professor Nunez stated that “AR errors are due to (1) limited electrode density and (2) incomplete electrode coverage (sampling only the upper part of head). If these errors were fully eliminated (only possible in detached heads), AR would provide the desired gold standard; that is, the nominal reference with respect to infinity” (Nunez 2010).

The fundamental assumption of AR is that the surface potential integral of a dipole in a volume conductor is zero, thus, the average potential of a dense and wide coverage electrode array is close to the ideal zero reference (Geselowitz 1998). However, such a zero potential integral assumption has been theoretically proved only for a spherical surface (Bertrand et al. 1985). In this communication, three counter-examples are given, which remind that the average may not always be zero for some specific surfaces.

## Demonstration

According to the theory of bio-electromagnetism, biological electric source is current source density, and for any current source density distribution $s(\vec{r})$, its potential satisfies Poisson’s equation

1 $${{\nabla }^{2}}\Phi \left( {\vec{r}} \right)\text{=}-s\left( {\vec{r}} \right)$$

And according to “Appendix A” section, the potential produced by such source $s(\vec{r})$ can be equivalently generated by a combination of multipole sources, i.e., monopole (point current source density), dipole, quadrupole, octapole etc. at the origin of a coordinate system. Among them the first monopole term (*0th*-order multipole) would be omitted due to the current conservation of a living system that requires the sum of the current source density inside a living system vanishes. The second dipole term (*1th*-order multipole) is consisted of a positive and a negative point current source density, and the other *l* ^th^-order multipoles with *l* > *1* are various complex linear combinations of dipoles (Wikswo et al. 1984, 1985). Furthermore, the linear relation between potential $\Phi$ and source $s(\vec{r})$ in Eq. (1) means that the principle of linear superposition is valid for EEG forward problem, thus we only need to check the potential integral over the surface of one dipole in a volume conductor.

### A Dipole in a Spherical Volume Conductor

For a dipole in a spherical volume conductor, it has been proved that the potential integral over the surface is zero (Bertrand et al. 1985). In fact, this conclusion is physically clear as the potential of a single dipole can be generated equivalently by a series of *l* ^th^-order multipole (*l* > = *1*) at the origin (“Appendix A” section). And the positive and negative potential of each *l* ^th^-order multipole (*l* > = *1*) always appear anti-symmetrically on the sphere surface, thus their integral must be zero. In another word, not only a dipole but also any combination of dipoles located anywhere in a sphere, the surface potential integral is zero. This fact is also valid for multilayer spherical model as the multipole expansion of neural electric sources in such a model is similar to the single sphere case (Yao 2000a, b).

### A Dipole in a Half-Space Volume Conductor

For a spherical volume conductor, if the radius *R* tends to infinity, the local surface of the sphere will evolve to the boundary plane of a half-space volume conductor. For such a model, any a dipole moment may be decomposed into two components, one is oriented parallel to the boundary plane, and the other perpendicular to the plane surface. Then, it is physically clear that the potential integral of the parallel one is zero as its positive and negative potential anti-symmetrically distribute on the plane. However, the potential integral of the perpendicular one will not be zero. The mathematical proof is shown in “Appendix B” section.

### A Dipole in a Homogenous Volume Conductor Except a Spherical Cave

Start from a half-space volume conductor, if the surface further bends to the opposite direction of the dipole, a special case may appear that a dipole locates in a homogenous volume conductor except a spherical cave. For such a case, if we assume a spherical coordinate system with origin at the center of the spherical cave with a dipole outside, the dipole moment may be decomposed into three components, one radial component perpendicular to the spherical surface, the other two oriented tangentially to the spherical surface. Similar to the half-space volume conductor model, the potential integrals of the two tangential components are zero, while that of the radial one is not zero. The mathematical proof is shown in “Appendix C” section, where the zero potential integral of a dipole in a spherical volume conductor is also proved passingly.

### A Dipole in a Homogeneous Semi-Sphere Volume Conductor

The above two models, a half-space and a homogeneous volume conductor with a spherical cave, looks not so like a head as the head would have a vivid almost closed surface. Here we further consider a homogeneous semi-sphere volume conductor.

First, we need the potential solution of a dipole in a homogeneous semi-sphere volume conductor, which must satisfies the Possion equation and the Neumann boundary condition. Suppose an actual dipole in the upper hemi-sphere of a spherical volume conductor, we may set a mirror dipole, both position and orientation mirrored, in the lower semi-sphere, and take the potential of the primary dipole in the sphere as $\Phi _{1}$, and that of the mirror dipole in the same sphere as $\Phi _{2}$, then we have both $\Phi _{1}$ and $\Phi {\text{ = }}\Phi _{1} {\text{ + }}\Phi _{2}$ satisfy the Possion equation in the upper semi-sphere and the Neumann boundary condition on the upper semi-spherical surface, both $\Phi _{1}$ and $\Phi {\text{ = }}\Phi _{1} {\text{ + }}\Phi _{2}$ satisfy the Possion equation in the lower semi-sphere and the Neumann boundary condition on the lower semi-spherical surface. For the circular plane, which divides the whole sphere into two semi-spheres, due to the orientation of the virtual dipole in the lower semi-sphere is mirror to the primary one, the Neumann boundary condition is also satisfied. Here the orientation mirror means that the two components parallel to the plane of the two dipoles are along the same direction, but the two components of them perpendicular to the plane are along opposite direction. These facts mean that $\Phi {\text{ = }}\Phi _{1} {\text{ + }}\Phi _{2}$ is the potential solution of a dipole in a semi-sphere volume conductor as it satisfies the Possion equation inside the semi-sphere conductor and the Neumann boundary condition on its whole surface. As we have the closed-form of $\Phi _{1}$ and $\Phi _{2}$ (Yao 2000a), we actually have the closed solution of a dipole in such a hemi-sphere volume conductor.

Now, let us check the potental integral over the whole surface of the semi-sphere conductor, we may consider the integrals over the hemi-spherical surface and the circular plane, separately. It is clear that the potential $\Phi _{2}$ at a point on the upper semi-spherical surface is the same as the potential $\Phi _{1}$ at a mirror point on the lower semi-spherical surface, thus the integral of $\Phi {\text{ = }}\Phi _{1} {\text{ + }}\Phi _{2}$ over the upper semi-spherical surface is equal to the integral of $\Phi _{1}$ over the whole sphere surface. According to “Appendix C” section, this integral is always zero (Bertrand et al. 1985). Now, for the potental integral of $\Phi {\text{ = }}\Phi _{1} {\text{ + }}\Phi _{2}$ over the circular plane, as the two dipoles mirror to each other about the circular plane, the potential $\Phi _{2}$ at a point on the circular plane is the same as the potential $\Phi _{1}$ at the same point, the sum $\Phi {\text{ = }}\Phi _{1} {\text{ + }}\Phi _{2}$ over the circular plane is the double of $\Phi _{1}$, the integral problem actually reduces to the potential integral of the potential $\Phi _{1}$ over the circular plane. The potential integral of a tangential dipole at the Cartesian coordinates $\left( 0,0,{{z}_{0}} \right)$ with the origin of the coordinate system at the center of the sphere is always zero as its potential distribution over the plane is anti-symmetrical, however, the mathematical deduction shown in “Appendix D” section indicates that the potential integral of a radial dipole at the Cartesian coordinates $\left( 0,0,{{z}_{0}} \right)$ is not zero but dependent on the radius *R* of the sphere and the value of $z_{0}$. Specifically, if the radius *R* tends to infinity, the integral reduces to the same as the half-space model shown in “Appendix B” section.

## Discussions

Though zero surface potential integral of a dipole in a conductor is proved only for a spherical surface (Bertrand et al. 1985), in current practice, “a consensus has emerged among researchers relying on data from dense electrode arrays that the use of an average reference may still be considered as the ‘gold standard’ for EEG analysis” (Srinivasan et al. 1998; Kayser and Tenke 2010). However, there is a 5.1% relative error between AR and true zero reference potential stubbornly there in a recent detailed simulation study with 256-channel EGI montage which covers almost the whole head (Liu et al. 2015). Such an error may not be totally attributed to the faulty montage and the discrete error of the boundary element method. In another recent detailed study, they found that the relative error between AR and true zero reference potential even increases with increasing sensor density for the tested electrode arrays from 21 to 128 channels (Chella et al. 2016). It means that array density may not be the most crucial factor.

What is the relation between the spherical model and half-plane model? As noted above, when the radius of a sphere extends to infinity, the local sphere surface tends to an infinite hemi-plane (the first counter-example).The theorem for spherical surface indicates that the integral over the whole sphere of the potential should exactly vanish (Bertrand et al. 1985; “Appendix C” section, Eq. (33)), but the integral over the half-plane may not vanish (“Appendix B” section, Eq. (10)). This fact means that the integral over the rest of the sphere should cancel the result for the plane. The reason is that the dipole potential at large distance L decreases as $1/L^{2}$, but the surface area of the integration at large distance behaves as $L^{2}$, the surface integral may be a finite value to cancel the plane integral.

In recent decades, there are some efforts aimed at improving the accuracy of AR by spherical spline interpolation (Junghofer et al. 1999), weighted average (Lemos and Fisch 1991), dynamic average (Orekhova et al. 2002) and generalized average (Carbonell et al. 2004). These methods are based on different additional assumptions, with each of them needs further comparative studies to test their performances.

Based on the above existed facts in literatures and the newly illustrated three counter examples, we’d like to ask, how reliable is the average potential over a head surface as EEG and ERP reference? In another word, is AR still a theoretical ‘gold standard’? Apparently, the above examples especially the semi-sphere model do shake its theoretical foundation that AR is not theoretically zero for general realistic head surface no matter what are the sensor density and coverage, and AR should not be taken as “gold standard” from now on. It means that we should be more open for other re-reference techniques and pay more attention to the potential fault of average reference in practice.

Is there any other potential ‘gold standard’ re-reference? In our opinion, the reference electrode standardization technique (REST) (Yao 2001) might be a latent one. REST utilizes the fact that the neural electric sources inside the brain are physically linked to the scalp recordings with any a reference, thus it is an intrinsic bridge, the Rosetta stone (Kayser and Tenke 2010), from one reference based recordings to the other. Furthermore, due to the non-uniqueness of the EEG inverse, same scalp recordings can be generated by different source configurations, where except the actual sources, all the other source configurations are equivalent sources of the actual sources in generating same scalp potential, and they can be used as the bridge from one reference recordings to the other, too. REST adopts equivalent distributed sources over the cortical surface as the bridge, thus only a linear inversion is needed (Yao 2001), and making REST easily realized. The equivalent distributed source model of any actual sources provides the theoretical foundation of REST(Yao 2000b; Yao and He 2003), and its accuracies were repeatedly confirmed by a serial simulation studies with comparison to AR and linked-ears etc. (Zhai et al. 2004; Marzetti et al. 2007; Qin et al. 2010; Liu et al. 2015; Chella et al. 2016). Its rationality in processing various real data were also proven step by step (Yao et al. 2005, 2007; Tian and Yao 2013; Bonfiglio et al. 2013; Xu et al. 2014; Chella et al. 2014, 2016; Kugiumtzis and Kimiskidis 2015), therefore it is quite valuable for further test and evaluation comparatively.

In summary, this short communication shows three examples revealing that the surface potential integral of a dipole in a volume conductor may not be zero, thus shaking the fundament assumption of the well-known average reference. We thus argue that further detailed comparative simulation studies and various real data evaluations among REST, AR and linked-ears etc. should be conducted in the near future to better confirm a timely ‘gold standard’ for various applications.

## Figures and Tables

**Fig. 1:**
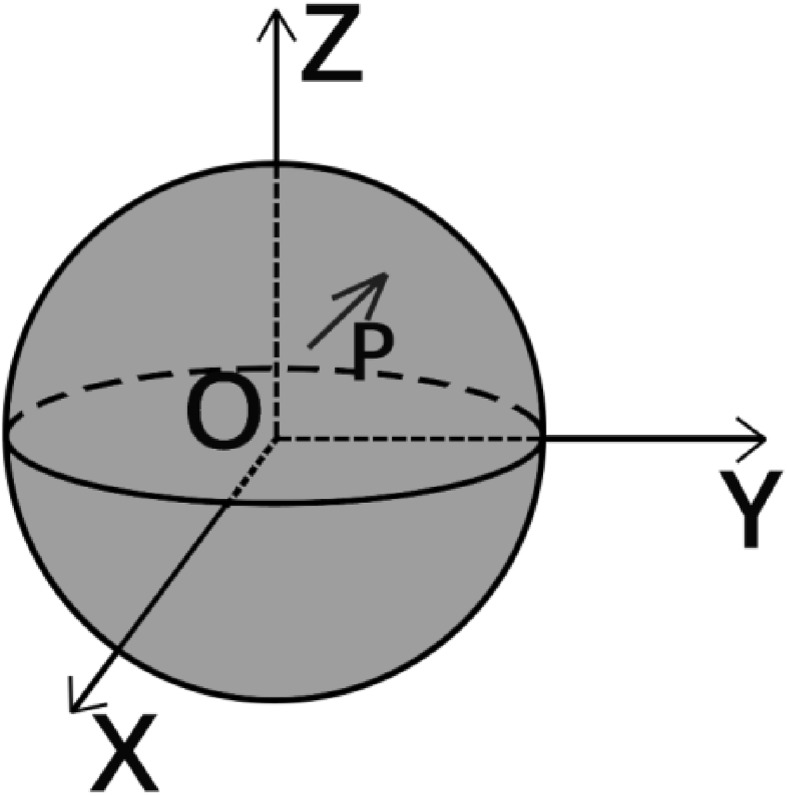
Illustration of a dipole in a spherical volume conductor. ${\vec{P}}$ is the dipole moment, the *grey area* is the spherical volume conductor

**Fig. 2:**
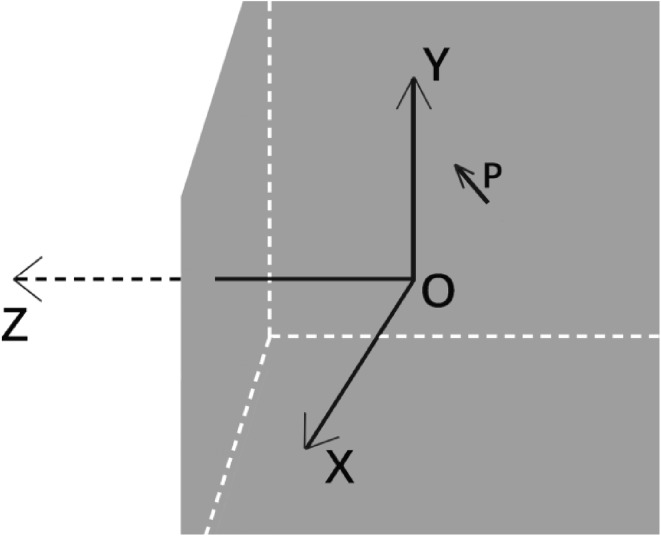
Illustration of a dipole in a half-space volume conductor. ${\vec{P}}$ is the dipole moment, the *grey area* is the volume conductor

**Fig. 3:**
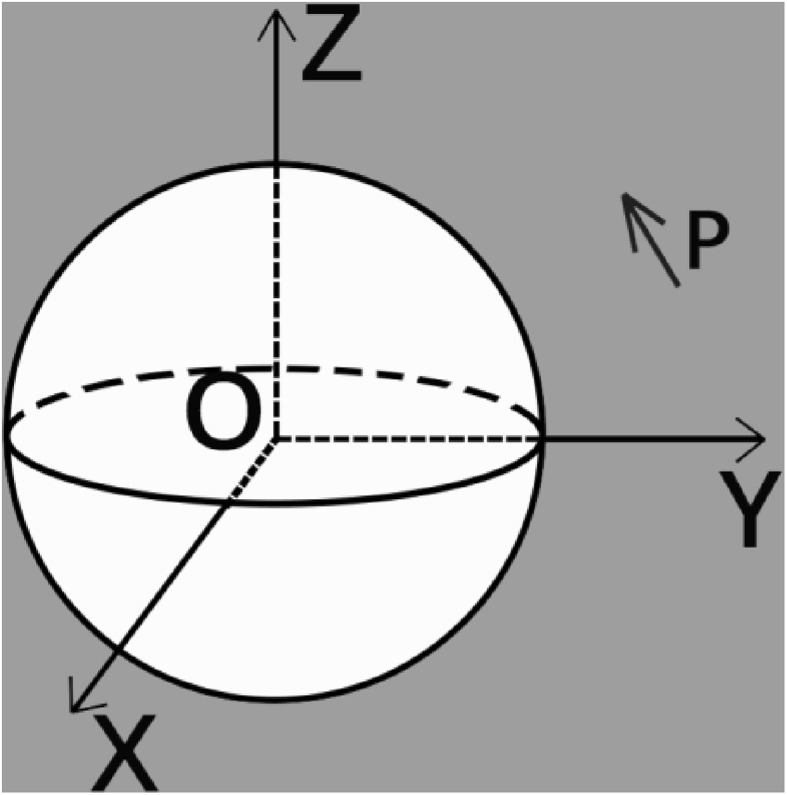
Illustration of a dipole in a volume conductor outside of a spherical cave. $\vec{P}$ is the dipole moment, the *grey area* is the volume conductor

## Appendix A: Potential Multipole Expansion of Any Electric Sources

According to bio-electromagnetic theory, biological electric source is current source density, and for any current source density distribution $s\left( {\vec{r}} \right)$, its potential satifies Poisson’s equation (Stratton 1941)

2 $${{\nabla }^{2}}\Phi \left( {\vec{r}} \right)\text{=}-s\left( {\vec{r}} \right)/\sigma$$

where $\vec{r}$ is the field point, $\sigma$ is the conductivity of the conductor (Geselowitz 1960). Equation (1) is a special case with unit conductivity. This equation is known to have a solution of the form

3 $$\Phi \left( {\vec{r}} \right)=\frac{1}{4\pi \sigma }\int_{v}{\frac{s\left( {{\vec{r}}'} \right)}{\left| \vec{r}-{\vec{r}}' \right|}{{d}^{3}}{\vec{r}}'}$$

If the source distribution region *v* can be bounded by a closed surface *S*, then the potential outside of *S* satisfies Laplace’s equation

4 $${{\nabla }^{2}}\Phi \left( {\vec{r}} \right)=0,\quad \vec{r}\notin v$$

For the area *r* > *R* with *R* as the maximum radius of *v*, Eq. (4) can be solved using spherical harmonic multipole expansion technique (Wikswo et al. 1984, 1985; Yao 2000b).

5 $$4\pi \sigma \Phi \left( {\vec{r}} \right)=\sum\limits_{l=0}^{\infty }{\sum\limits_{m=0}^{l}{\frac{1}{{{r}^{l+1}}}}}\left( a_{l}^{m}\cos m\phi +b_{l}^{m}\sin m\phi \right)P_{l}^{m}\left( \cos \theta \right),r>R$$

where $P_{l}^{m}\left( \cos \theta \right)$ being the associated Lgendre function of the first kind, the coefficients may be represented as

6 $$a_{l}^{m}+jb_{l}^{m}={{\varepsilon }_{\text{m}}}\frac{\left( l-\text{m} \right)\text{!}}{\left( l+m \right)!}\int_{0}^{2\pi }{{{e}^{jm{\phi }'}}}d{\phi }'\int_{0}^{\pi }{P_{l}^{m}\left( \cos {\theta }' \right)\sin {\theta }'d{\theta }'}\int_{0}^{\text{R}}{s\left( {{\vec{r}}'} \right){{\left( {{r}'} \right)}^{l+2}}d{r}'}$$

where $\varepsilon$ is the Neumann factor:$\varepsilon = 1$ for m = 0,and $\varepsilon = 2$ for ${\text{m}} \ne 0.$ $b_{1}^{0} = 0$ for all *l*, $j = \sqrt { - 1}$. The first term $a_{0}^{0}$ corresponds to monopole (*l* = *0*) term, it would be omitted due to the current conservation of a living system that requires the total sum of the current source densities inside a living system vanishes. There are three dipole components $\left( a_{1}^{0},a_{1}^{1}\text{,b}_{1}^{1} \right)$ (*l* = *1*), and (*2l* + *1*) components for the following *l* ^th^ order multipole.

Equation (2)–(6) show us that the potential produced by any sources $s\left( {\vec{r}} \right)$ in the head can be equivalently generated by a combination of dipole, quadrupole, octapole etc., at the origin of a coordinate system.

## Appendix B: A Dipole in a Half-Space Volume Conductor

For a dipole in a sphere (Fig. 1), with the Cartesian coordinate origin at the center, the potential on the sphere surface is (Yao 2000a)

7 $$\Phi =\frac{\vec{P}\cdot }{4\pi \sigma }\left\{ 2\frac{{{{\vec{r}}}_{p}}}{r_{p}^{3}}+\frac{1}{{{R}^{2}}{{r}_{p}}}\left[ \vec{R}+\frac{\vec{R}{{r}_{0}}\cos \varphi -R{{{\vec{r}}}_{0}}}{R+{{r}_{p}}-{{r}_{0}}\cos \varphi } \right] \right\}$$

where $\vec{R}$ and $\vec{r}_{0}$ are the field point (on the sphere surface) and dipole source location, $r_{{\text{p}}} \left( {{{\vec{r}}}_{{\text{p}}} {\text{ = }}\vec{R} - {{\vec{r}}}_{0} } \right)$ and $\varphi$ are the distance and angle between $\vec{R}$ and $\vec{r}_{0}$, respectively. ${{\vec{P}}}$ is the dipole moment, $\sigma$ is the conductivity of the volume conductor. *R* is the radius of the sphere. Apparently, when *R* extends to infinity, $R \to \infty$, the formula simplifies to

8 $$\Phi =\frac{2}{4\pi \sigma }\frac{\vec{P}\cdot {{{\vec{r}}}_{p}}}{r_{p}^{3}}=\frac{2}{4\pi \sigma }\frac{\vec{P}\cdot \left( \vec{R}-{{{\vec{r}}}_{0}} \right)}{|{{{\vec{R}}}_{{}}}-{{{\vec{r}}}_{0}}{{|}^{3}}}$$

It is the potential formula for a dipole in a half-space volume conductor (Fig. 2). This formula can also be derived by mirror image source method in electromagnetic field theory. Now to further simplify the following deduction, we suppose that the Cartesian coordinate origin is at the source point, then $r_{0} {\text{ = }}0$, and ${{\vec{r}}_{p}}\text{=}\vec{R}-{{\vec{r}}_{0}}\text{=x}{{\vec{e}}_{x}}+y{{\vec{e}}_{y}}+z{{\vec{e}}_{z}},$ $\vec{P}\text{=}{{\text{P}}_{x}}{{\vec{e}}_{x}}+{{P}_{y}}{{\vec{e}}_{y}}+{{P}_{z}}{{\vec{e}}_{z}}$, where ${{\vec{e}}_{x}}\,,{{\vec{e}}_{y}}\,,{{\vec{e}}_{z}}$ are the three unit vectors of the Cartesian coordinate system. Then we have

9 $$\Phi =\frac{2}{4\pi \sigma }\frac{{{P}_{z}}z+{{P}_{x}}x\text{+}{{P}_{\text{y}}}y}{{{\left( {{x}^{2}}+{{y}^{2}}+{{z}^{2}} \right)}^{3/2}}}$$

Now, let’s consider the potential integrals of the three dipole components separately. For the z-axis component, the integral over the boundary plane (z = constant, Fig. 2) is

10 $$\begin{gathered} \Phi _{z} = \frac{2}{{4\pi \sigma }}\int\limits_{{ - \infty }}^{\infty } {\int\limits_{{ - \infty }}^{\infty } {\frac{{P_{z} z}}{{\left( {x^{2} + y^{2} + z^{2} } \right)^{{3/2}} }}} dxdy} \hfill \\ = \frac{{2P_{z} \cdot z}}{{4\pi \sigma }}\int\limits_{0}^{{2\pi }} {\int\limits_{0}^{\infty } {\frac{1}{{\left( {r_{{xy}} ^{2} + z^{2} } \right)^{{3/2}} }}} r_{{xy}} d\theta dr_{{xy}} } \hfill \\ = \frac{{P_{z} }}{\sigma } \hfill \\ \end{gathered}$$

For the x-axis component, the integral over the surface plane (z = constant) is

11 $$\begin{gathered} \Phi _{x} = \frac{2}{{4\pi \sigma }}\int\limits_{{ - \infty }}^{\infty } {\int\limits_{{ - \infty }}^{\infty } {\frac{{P_{x} x}}{{(x^{2} + y^{2} + z^{2} )^{{3/2}} }}} dxdy} \hfill \\ = \frac{{2P_{x} }}{{4\pi \sigma }}\int\limits_{0}^{{2\pi }} {\int\limits_{0}^{\infty } {\frac{x}{{(r_{{xy}} ^{2} + z^{2} )^{{3/2}} }}} r_{{xy}} d\theta dr_{{xy}} } = \frac{{2P_{x} }}{{4\pi \sigma }}\int\limits_{0}^{{2\pi }} {\int\limits_{0}^{\infty } {\frac{{r_{{xy}} \cos \theta }}{{(r_{{xy}} ^{2} + z^{2} )^{{3/2}} }}} r_{{xy}} d\theta dr_{{xy}} } \hfill \\ = \int\limits_{0}^{{2\pi }} {\cos \theta d\theta } \frac{{2P_{x} }}{{4\pi \sigma }}\int\limits_{0}^{\infty } {\frac{{r_{{xy}} ^{2} }}{{(r_{{xy}} ^{2} + z^{2} )^{{3/2}} }}} dr_{{xy}} = 0 \hfill \\ \end{gathered}$$

Similarly, the potential integral of y-axis component is also zero.

These results are physically very clear. For the z-axis component, only positive or negative potential of the dipole is on the plane, while for the x- or y-axis component, the positive and negative potential are anti-symmetrically distributed on the plane, thus their sum is zero.

## Appendix C: A Dipole in a Homogenous Volume Conductor Except a Spherical Cave

In general, the potential of a dipole in an infinite volume conductor is

12 $${{\Phi }_{\infty }}=\frac{1}{4\pi \sigma }\frac{\vec{P}\cdot {{{\vec{r}}}_{p}}}{r_{p}^{3}},\quad {{\vec{r}}_{p}}=\vec{r}-{{\vec{r}}_{0}},\quad {{r}_{p}}=\sqrt{{{r}^{2}}+r_{0}^{2}-\text{2r}{{\text{r}}_{0}}\text{cos}\varphi }$$

where ${{\vec{r}}}\left( {{\text{r,}}\theta ,\phi } \right)$ and ${{\vec{r}}_{0}}\left( {{\text{r}}_{0}},{{\theta }_{0}},{{\phi }_{0}} \right)$ are the field point and dipole source location, $r_{{\text{p}}}$ and $\varphi$ are the distance and angle between ${{\vec{r}}}$ and $\vec{r}_{0}$, respectively. Equation (12) also may be written as (Yao 2000b; Stratton 1941):

13 $$\Phi _{\infty } = \frac{1}{{4\pi \sigma }}{{\vec{P}}} \cdot \nabla _{{r_{0} }} \left( {\frac{1}{{r_{p} }}} \right) = \frac{1}{{4\pi \sigma }}\left\{ {P_{r} \frac{\partial }{{\partial r_{0} }} + P_{\phi } \frac{1}{{r_{0} \sin \theta _{0} }}\frac{\partial }{{\partial \phi _{0} }} + P_{\theta } \frac{1}{{r_{0} }}\frac{\partial }{{\partial \theta _{0} }}} \right\}\frac{1}{{r_{p} }}$$

Here $1/{r}_{p}$ may be expanded as a series. For ${\text{r> r}}_{0}$, we have

14 $$\frac{1}{{{r}_{p}}}=\frac{1}{{{r}_{{}}}}\frac{1}{\sqrt{1+{{\left( \frac{{{r}_{0}}}{r} \right)}^{2}}-\text{2}\frac{{{\text{r}}_{0}}}{r}\text{cos}\varphi }}\text{=}\sum\limits_{l=0}^{\infty }{\sum\limits_{m=0}^{l}{\frac{r_{0}^{l}}{{{r}^{l+1}}}}}\left( 2-\delta _{m}^{0} \right)\frac{\left( l-m \right)!}{\left( l+m \right)!}P_{l}^{m}\left( \cos {{\theta }_{0}} \right)P_{l}^{m}\left( \cos \theta \right)\cos m\left( \phi -{{\phi }_{0}} \right)$$

For ${\text{r < r}}_{0}$, we have

15 $$\frac{1}{{{r}_{p}}}=\frac{1}{{{r}_{0}}}\frac{1}{\sqrt{1+{{\left( \frac{r}{{{r}_{0}}} \right)}^{2}}-\text{2}\frac{\text{r}}{{{r}_{0}}}\text{cos}\varphi }}\text{=}\sum\limits_{l=0}^{\infty }{\sum\limits_{m=0}^{l}{\frac{r_{{}}^{l}}{{{r}_{0}}^{l+1}}}}\left( 2-\delta _{m}^{0} \right)\frac{\left( l-m \right)!}{\left( l+m \right)!}P_{l}^{m}\left( \cos {{\theta }_{0}} \right)P_{l}^{m}\left( \cos \theta \right)\cos m\left( \phi -{{\phi }_{0}} \right)$$

Equations (14) and (15) are quite similar except the roles of *r* and ${\text{r}}_{0}$ exchanged. $P_{l}^{m}$ is the associate Legendre function. Taking into consideration of these Legendre function relations:

16 $$\frac{\partial P_{l}^{m}}{\partial {{\theta }_{0}}}=-\frac{1}{2}\left[ \left( l-m+1 \right)\left( l+m \right)P_{l}^{m-1}\left( \cos {{\theta }_{0}} \right)-P_{l}^{m+1}\left( \cos {{\theta }_{0}} \right) \right],\frac{\partial {{P}_{l}}}{\partial {{\theta }_{0}}}=P_{l}^{1}\left( \cos {{\theta }_{0}} \right)$$

Invoking Eq. (16), Eq. (15) into Eq. (13), we have the following equations for ${\text{r> r}}_{0}$ case.

17 $$\begin{aligned} \Phi _{\infty } &= \sum\limits_{{l = 0}}^{\infty } {\sum\limits_{{m = 0}}^{l} {\frac{{K_{l}^{m} \left( {\vec{P}} \right)}}{{r^{{l + 1}} }}\left( {Z_{l}^{m} \cos m\phi + Y_{l}^{m} \sin m\phi } \right)} P_{l}^{m} \left( {\cos \theta } \right)} \\ &= \sum\limits_{{l = 0}}^{\infty } {\sum\limits_{{m = 0}}^{l} {\frac{{K_{l}^{m} \left( {\vec{P}} \right)}}{{r^{{l + 1}} }}S_{l}^{m} \left( {\vec{P},\theta ,\phi } \right)} } \end{aligned}$$

where

18 $$S_{l}^{m}\left( \vec{P},\theta ,\phi \right)=\left( Z_{l}^{m}\cos m\phi +Y_{l}^{m}\sin m\phi \right)P_{l}^{m}\left( \cos \theta \right)$$

19 $$K_{l}^{m}\left( {\vec{P}} \right)=\frac{r_{0}^{l-1}}{4\pi \sigma }\left( 2-\delta _{m}^{0} \right)\frac{\left( l-m \right)!}{\left( l+m \right)!}$$

20 $$\begin{gathered} Z_{l}^{m} {\text{ = }}lp_{r} P_{l}^{m} \left( {\cos \theta _{0} } \right)\cos m\varphi _{0} - \frac{{mp_{\phi } }}{{\sin \theta _{0} }}P_{l}^{m} \left( {\cos \theta _{0} } \right)\sin m\varphi _{0} - \hfill \\ \frac{{p_{\theta } }}{2}\left( {\left( {l - m + 1} \right)\left( {l + m} \right)P_{l}^{{m - 1}} \left( {\cos \theta _{0} } \right) - P_{l}^{{m + 1}} \left( {\cos \theta _{0} } \right)} \right)\cos m\varphi _{0} \hfill \\ \end{gathered}$$

21 $$\begin{gathered} Y_{l}^{m} {\text{ = }}lp_{r} P_{l}^{m} \left( {\cos \theta _{0} } \right)\sin m\varphi _{0} + \frac{{mp_{\phi } }}{{\sin \theta _{0} }}P_{l}^{m} \left( {\cos \theta _{0} } \right)\cos m\varphi _{0} - \hfill \\ \frac{{p_{\theta } }}{2}\left( {\left( {l - m + 1} \right)\left( {l + m} \right)P_{l}^{{m - 1}} \left( {\cos \theta _{0} } \right) - P_{l}^{{m + 1}} \left( {\cos \theta _{0} } \right)} \right)\sin m\varphi _{0} \hfill \\ \end{gathered}$$

Considering the difference between Eq. (14) with ${\text{r> r}}_{0}$ and Eq. (15) with ${\text{r < r}}_{0}$, and the derivtives related to ${\text{r}}_{0}$ in Eq. (13), we have the following equation for ${\text{r < r}}_{0}$ case.

22 $$\begin{aligned} \Phi _{\infty } &= \sum\limits_{{l = 0}}^{\infty } {\sum\limits_{{m = 0}}^{l} {\frac{{C_{l}^{m} \left( {\vec{P}} \right){\text{r}}^{l} }}{{r_{0}^{{{\text{l + 1}}}} }}\left( {G_{l}^{m} \cos m\phi + H_{l}^{m} \sin m\phi } \right)} P_{l}^{m} \left( {\cos \theta } \right)} \\ &= \sum\limits_{{l = 0}}^{\infty } {\sum\limits_{{m = 0}}^{l} {\frac{{C_{l}^{m} \left( {\vec{P}} \right){\text{r}}^{l} }}{{r_{0}^{{{\text{l + 1}}}} }}T_{l}^{m} \left( {\vec{P},\theta ,\phi } \right)} } \end{aligned}$$

where

23 $$T_{l}^{m}\left( \vec{P},\theta ,\phi \right)=\left( G_{l}^{m}\cos m\phi +H_{l}^{m}\sin m\phi \right)P_{l}^{m}\left( \cos \theta \right)$$

24 $$C_{l}^{m}\left( {\vec{P}} \right)=\frac{\text{r}_{{}}^{l}}{4\pi \sigma {{r}_{0}}}\left( 2-\delta _{m}^{0} \right)\frac{\left( l-m \right)!}{\left( l+m \right)!}$$

25 $$\begin{gathered} G_{l}^{m} = - \left( {l + 1} \right)p_{r} P_{l}^{m} \left( {\cos \theta _{0} } \right)\cos m\phi _{0} - \frac{{mp_{\phi } }}{{\sin \theta _{0} }}P_{l}^{m} \left( {\cos \theta _{0} } \right)\sin m\phi _{0} - \hfill \\ \frac{{p_{\theta } }}{2}\left( {\left( {l - m + 1} \right)\left( {l + m} \right)P_{l}^{{m - 1}} \left( {\cos \theta _{0} } \right) - P_{l}^{{m + 1}} \left( {\cos \theta _{0} } \right)} \right)\cos m\varphi _{0} \hfill \\ \end{gathered}$$

26 $$\begin{gathered} H_{l}^{m} = - \left( {l + 1} \right)p_{r} P_{l}^{m} \left( {\cos \theta _{0} } \right)\sin m\phi _{0} + \frac{{mp_{\phi } }}{{\sin \theta _{0} }}P_{l}^{m} \left( {\cos \theta _{0} } \right)\cos m\phi _{0} - \hfill \\ \frac{{p_{\theta } }}{2}\left( {\left( {l - m + 1} \right)\left( {l + m} \right)P_{l}^{{m - 1}} \left( {\cos \theta _{0} } \right) - P_{l}^{{m + 1}} \left( {\cos \theta _{0} } \right)} \right)\sin m\varphi _{0} \hfill \\ \end{gathered}$$

Now, if there is a spherical boundary to have the dipole inside the spherical volume conductor (Fig. 1) or to have the dipole outside the spherical case (Fig. 3), according to the uniqueness theory of the Neumann boundary value problem (Yao 2000a), the potential of the dipole inside/outside the sphere of radius R may be obtained by adding to Eq. (17)/(22) a solution $\Phi _{i}$ of Laplace’s equation which has no poles inside/outside the spherical region, respectively, and make $\partial \Phi /\partial \text{r}=\partial \left( {{\Phi }_{\infty }}+{{\Phi }_{i}} \right)/\partial \text{r=}0$ at *r* = *R*.

The final results would be

27 $$\Phi =\sum\limits_{l=1}^{\infty }{\sum\limits_{m=0}^{l}{\left( \frac{1}{{{r}^{l+1}}}\text{+}\frac{{{r}^{l}}}{{{R}^{2l+1}}}\frac{l+1}{l} \right)K_{l}^{m}\left( {\vec{P}} \right)S_{l}^{m}\left( \vec{P},\theta ,\phi \right)}},\quad \text{for}\,\,R>\,=r>{{r}_{0}}$$

And

28 $$\Phi =\sum\limits_{l=1}^{\infty }{\sum\limits_{m=0}^{l}{\left( {{r}^{l}}\text{+}\frac{l}{l+1}\frac{{{R}^{2l+1}}}{{{r}^{l+1}}} \right)C_{l}^{m}\left( {\vec{P}} \right)T_{l}^{m}\left( \vec{P},\theta ,\phi \right),\quad \text{for}\,}}\,R=<r<{{r}_{0}}$$

Now consider the potential integral over the spherical surface (*r* = *R*),

29 $$\text{V}=\int\limits_{0}^{\pi }{\int\limits_{0}^{2\pi }{\Phi {{\text{R}}^{2}}\sin \theta d\theta }}d\phi$$

It can be processed as two parts of $S_{l}^{m}$ or $T_{l}^{m}$, respectively. The first term involves

30 $$\begin{gathered} {\text{V}}_{1} = \int\limits_{0}^{\pi } {\int\limits_{0}^{{2\pi }} {\cos {\text{m}}\phi P_{l}^{{\text{m}}} \left( {\cos \theta } \right)\sin \theta d\theta {\text{d}}\phi } } \hfill \\ = \int\limits_{0}^{\pi } {P_{l}^{{\text{m}}} \left( {\cos \theta } \right)\sin \theta d\theta \int\limits_{0}^{{2\pi }} {\cos {\text{m}}\phi } } d\phi \hfill \\ = \left\{ \begin{gathered} 2\pi \int\limits_{0}^{\pi } {P_{l}^{{\text{0}}} \left( {\cos \theta } \right)\sin \theta d\theta } {\text{ = }} - 2\pi \int\limits_{0}^{\pi } {P_{l}^{{\text{0}}} \left( {\cos \theta } \right){\text{d}}cos\theta } \hfill \\ 0,\quad {\text{m}} \ne 0 \hfill \\ \end{gathered} \right. \hfill \\ {\text{ = }}\left\{ \begin{gathered} 2\pi \int\limits_{{ - 1}}^{1} {P_{l}^{{\text{0}}} \left( {\text{u}} \right){\text{du}}} {\text{ = }}2\pi \int\limits_{{ - 1}}^{1} {P_{l}^{{\text{0}}} \left( {\text{u}} \right)P_{0}^{{\text{0}}} \left( {\text{u}} \right){\text{du = 4}}} \pi ,\quad {\text{l = }}m = 0 \hfill \\ 0,\quad other\,cases \hfill \\ \end{gathered} \right. \hfill \\ \end{gathered}$$

where we utilized

$$\int\limits_{0}^{{2\pi }} {\cos {\text{m}}\phi } {\text{d}}\phi {\text{ = }}\left\{ \begin{gathered} \int\limits_{0}^{{2\pi }} {{\text{d}}\phi } {\text{ = }}2\pi ,\quad m = 0 \hfill \\ \frac{1}{m}\int\limits_{0}^{{2\pi }} {\left( { - d\sin m\phi } \right) = 0,\quad m \ne 0} \hfill \\ \end{gathered} \right.$$

The second term involves

31 $$\begin{gathered} {\text{V}}_{2} = \int\limits_{0}^{\pi } {\int\limits_{0}^{{2\pi }} {\sin {\text{m}}\phi P_{l}^{{\text{m}}} \left( {\cos \theta } \right)\sin \theta d\theta {\text{d}}\phi } } \hfill \\ = \int\limits_{0}^{\pi } {P_{l}^{{\text{m}}} \left( {\cos \theta } \right)\sin \theta d\theta \int\limits_{0}^{{2\pi }} {\sin {\text{m}}\phi } } d\phi \hfill \\ = 0 \hfill \\ \end{gathered}$$

For the case ${\text{r}}_{0} < {\text{R}}$, dipole in the spherical volume conductor, even for l = m = 0, according to Eqs. (13), (15), (16) and ${P}_{0}^{0}(x)=1$, $\text{Y}_{0}^{0}=Z_{0}^{0}=0$, it means that there is no monopole in the mutipole expansion series of a dipole. Combing these facts with Eqs. (30) and (31), it means

32 $$\int\limits_{0}^{\pi }{\int\limits_{0}^{2\pi }{S_{l}^{m}\left( \vec{P},\theta ,\phi \right)\sin \theta d\theta }\text{=}0}$$

And

33 $$\text{V}=\int\limits_{0}^{\pi }{\int\limits_{0}^{2\pi }{\Phi {{\text{R}}^{2}}\sin \theta d\theta }}\text{=}0$$

Equation (33) confirms the statement that the potential integral over a spherical surface is zero when a dipole inside a spherical volume conductor (Bertrand et al. 1985). This fact is reasonable as the equivalent multipoles at the coordinate origin of a dipole is consisted of dipole, quadrupole, octapole etc, there is no monopole which may produce a constant non-zero poential over the whole spherical surface, and the other *l* ^th^-order multipole all produce a anti-symmetrical positive and negative potential distriution over the surface, thus their integral is definitely zero.

For the case ${\text{R < r}}_{0}$, a dipole outside a spherical cave, for l = m = 0, according to Eqs. (25) and (26), we have

34 $$\text{G}_{0}^{0}=-{{P}_{r}},H_{0}^{0}=0$$

Invoking Eq. (30), Eq. (31) into Eqs. (23–26, 28), we have

35 $$\text{V}=\int\limits_{0}^{\pi }{\int\limits_{0}^{2\pi }{\Phi {{\text{R}}^{2}}\sin \theta d\theta d\phi }}\text{=}-\frac{4{{P}_{r}}{{R}^{2}}}{\sigma {{\text{r}}_{0}}}$$

This fact means that for a dipole outside a spherical cave, the radial component of the dipole may evoking an equivlaent monopole term at the origin, its surface potential integral is not zero except the dipole is quite far away (${{r}_{0}}\to \infty$). Apparently, for a ${{\vec{P}}}_{r}$ oriented outside the spherical cave, the negative pole of the dipole close to the surface, the surface integral is negative, for an opposite case, the integral is positive.

## Appendix D: The Potential Integral Over the Circular Plane of a Dipole in a Homogenous Semi-Sphere Volume Conductor

As explained in the above “A Dipole in a Homogeneous Semi-Sphere Volume Conductor” section, the potential $\Phi$ on the circular plane of a dipole in a homogeneous semi-sphere volume conductor is the double of the potential on the same plane of the same dipole in a homogeneous sphere volume conductor, thus we may just work on the potential integral of the potential $\Phi _{1}$ over the circular plane.

In general, for a dipole in a homogenous spherical volume conductor, the potential at anywhere in the sphere is (Yao 2000a)

36 $${{\Phi }_{1}}=\frac{\vec{P}\cdot }{4\pi \sigma }\left\{ \frac{\vec{r}-{{{\vec{r}}}_{0}}}{r_{p}^{3}}+\frac{\left( \vec{r}-\frac{{{r}^{2}}}{{{R}^{2}}}{{{\vec{r}}}_{0}} \right)}{{{R}^{3}}r_{pi}^{3}}+\frac{1}{{{R}^{3}}{{r}_{pi}}}\left[ \vec{r}+\frac{\vec{r}\frac{{{r}_{0}}r}{{{R}^{2}}}\cos \phi -\frac{{{r}^{2}}}{{{R}^{2}}}{{{\vec{r}}}_{0}}}{{{r}_{pi}}+1-\frac{{{r}_{0}}r}{{{R}^{2}}}\cos \phi } \right] \right\}$$

where the Cartesian coordinates of the field point $\vec{r}$ and the position $\vec{r}_{0}$ of the dipole with moment $\vec{p}$ are (x, y, z) and $\left( {{x}_{0}},{{y}_{0}},{{z}_{0}} \right)$, respectively, and the length of the displacement ${{\vec{r}}_{p}}=\vec{r}-{{\vec{r}}_{0}}$ between these two points are ${{\text{r}}_{p}}\text{=}{{\left( {{\text{r}}^{2}}\text{+r}_{0}^{2}-\text{2r}{{\text{r}}_{0}}\cos \phi \right)}^{1/2}}$, $\phi$ is the angle between $\vec{r}$ and $\vec{r}_{0}$, R is the radius of the sphere, $\sigma$ is the conductivity of the volume conductor. Where ${{\text{r}}_{pi}}\text{=}{{\left( \text{1+}{{\left( \text{r}{{\text{r}}_{0}}\text{/}{{\text{R}}^{2}} \right)}^{2}}-\text{2r}{{\text{r}}_{0}}\text{/}{{\text{R}}^{2}}\cos \phi \right)}^{1/2}}$. Here, as an example, we specifically consider a dipole at Cartesian coordinates $\left( {{x}_{0}},{{y}_{0}},{{z}_{0}} \right)\text{=}\left( 0,0,{{z}_{0}} \right)$ and positively oriented along z–axis with ${{\vec{P} = P}}_{x} {{\vec{e}}}_{x} + {\text{P}}_{y} {{\vec{e}}}_{y} + {\text{P}}_{z} {{\vec{e}}}_{z} = {\text{P}}_{z} {{\vec{e}}}_{z}$, where $\left( {{\text{P}}_{x}},{{\text{P}}_{y}}\text{,}{{\text{P}}_{z}} \right)$ are the three components of the dipole moment ${{\vec{P}}}$ in accordance with the three unit vectors $\left( {{{\vec{e}}}_{x}},{{{\vec{e}}}_{y}},{{{\vec{e}}}_{z}} \right)$ of the Cartesian coordinate system with origin at the center of the sphere. For the potential on the circular plane (z = 0), we have $\phi ={{90}^{\text{o}}}$ and $\vec{r}\text{=}\left( \text{x}{{{\vec{e}}}_{x}},y{{{\vec{e}}}_{y}},z{{{\vec{e}}}_{z}} \right)=\left( \text{x}{{{\vec{e}}}_{x}},y{{{\vec{e}}}_{y}},0{{{\vec{e}}}_{z}} \right)$.

For this special case, we get the following simplified formula Eq. (37) from the above Eq. (36).

37 $$\Phi =2{{\Phi }_{1}}\text{=}-2\frac{{{P}_{\text{z}}}}{4\pi \sigma }\left[ \frac{{{z}_{0}}}{r_{p}^{3}}+\frac{{{r}^{2}}{{z}_{0}}}{{{R}^{5}}r_{pi}^{3}}\text{+}\frac{{{r}^{2}}{{z}_{0}}}{{{R}^{5}}{{r}_{pi}}\left( {{r}_{pi}}+1 \right)} \right]$$

where $$\Phi$$ is the total potential of the dipole and its mirror, the double of $$\Phi _{1} ,r_{p} = \left( {r^{2} + {\text{z}}_{0}^{2} } \right)^{{{\raise0.7ex\hbox{$1$} \!\mathord{\left/ {\vphantom {1 2}}\right.\kern-\nulldelimiterspace} \!\lower0.7ex\hbox{$2$}}}}$$, $${{r}^{2}}\text{=}{{\text{x}}^{2}}+{{y}^{2}}$$, $${{r}_{pi}}={{\left( 1+{{\left( {{r}_{0}}r/{{R}^{2}} \right)}^{2}} \right)}^{{}^{1}\!\!\diagup\!\!{}_{2}\;}}\text{=1/}{{\text{R}}^{2}}{{\left( {{R}^{4}}+{{\left( {{z}_{0}}r \right)}^{^{2}}} \right)}^{^{{}^{1}\!\!\diagup\!\!{}_{2}\;}}}.$$

Then the integral over the circular plane is

38 $$\text{I}=\int\limits_{0}^{2\pi }{\int\limits_{0}^{R}{\Phi }rd\theta }dr=-\frac{{{P}_{\text{z}}}}{\sigma }\int\limits_{0}^{R}{\left[ \frac{{{z}_{0}}}{r_{p}^{3}}+\frac{{{r}^{2}}{{z}_{0}}}{{{R}^{5}}r_{pi}^{3}}\text{+}\frac{{{r}^{2}}{{z}_{0}}}{{{R}^{5}}{{r}_{pi}}\left( {{r}_{pi}}+1 \right)} \right]}r\text{dr}=-\frac{{{P}_{\text{z}}}}{\sigma }\left[ {{I}_{1}}+{{I}_{2}}+{{I}_{3}} \right]$$

Specifically, if the dipole is just located on the circular plane $\left( {z_{0} = 0} \right)$, the potential shown by Eq. (37) and the integral shown in Eq. (38) are all zero. However, this case is not the situation that we concerned that a dipole is in a semi-sphere volume conductor with $z_{0}> 0$.

Now we check each item in Eq. (38) for $z_{0}> 0$,we have

39 $${{\text{I}}_{1}}={{z}_{0}}\int\limits_{0}^{R}{\frac{1}{{{\left( {{r}^{2}}+\text{z}_{0}^{2} \right)}^{{}^{3}\!\!\diagup\!\!{}_{2}\;}}}}r\text{dr}=\frac{{{z}_{0}}}{2}\int\limits_{0}^{R}{\frac{1}{{{\left( {{r}^{2}}+\text{z}_{0}^{2} \right)}^{{}^{3}\!\!\diagup\!\!{}_{2}\;}}}}\text{d}{{\text{r}}^{2}}\text{=}-\frac{{{z}_{0}}}{{{\left( {{\text{R}}^{2}}+\text{z}_{0}^{2} \right)}^{{}^{1}\!\!\diagup\!\!{}_{2}\;}}}+1$$

and

40 $$\begin{gathered} {\text{I}}_{2} {\text{ = }}\int\limits_{0}^{R} {\frac{{r^{2} z_{0} }}{{R^{3} \left( {R^{4} + \left( {z_{0} r} \right)^{2} } \right)^{{{\raise0.7ex\hbox{$3$} \!\mathord{\left/ {\vphantom {3 2}}\right.\kern-\nulldelimiterspace} \!\lower0.7ex\hbox{$2$}}}} }}} r{\text{dr}} = \frac{{z_{0} }}{{2R^{3} }}\int\limits_{0}^{R} {\frac{{r^{2} }}{{\left( {R^{4} + {\text{z}}_{0}^{2} r^{2} } \right)^{{{\raise0.7ex\hbox{$3$} \!\mathord{\left/ {\vphantom {3 2}}\right.\kern-\nulldelimiterspace} \!\lower0.7ex\hbox{$2$}}}} }}} {\text{dr}}^{2} \hfill \\ = \frac{{z_{0} }}{{2{\text{z}}_{0}^{2} R^{3} }}\int\limits_{0}^{R} {\left[ {\frac{{R^{4} + {\text{z}}_{0}^{2} r^{2} }}{{\left( {R^{4} + {\text{z}}_{0}^{2} r^{2} } \right)^{{{\raise0.7ex\hbox{$3$} \!\mathord{\left/ {\vphantom {3 2}}\right.\kern-\nulldelimiterspace} \!\lower0.7ex\hbox{$2$}}}} }} - \frac{{R^{4} }}{{\left( {R^{4} + {\text{z}}_{0}^{2} r^{2} } \right)^{{{\raise0.7ex\hbox{$3$} \!\mathord{\left/ {\vphantom {3 2}}\right.\kern-\nulldelimiterspace} \!\lower0.7ex\hbox{$2$}}}} }}} \right]} {\text{dr}}^{2} \hfill \\ = \frac{{z_{0} }}{{2{\text{z}}_{0}^{2} R^{3} }}\left\{ {\frac{2}{{{\text{z}}_{0}^{2} }}\left[ {\left( {R^{4} + {\text{z}}_{0}^{2} R^{2} } \right)^{{{\raise0.7ex\hbox{$1$} \!\mathord{\left/ {\vphantom {1 2}}\right.\kern-\nulldelimiterspace} \!\lower0.7ex\hbox{$2$}}}} - R^{2} } \right] + \frac{{2R^{4} }}{{{\text{z}}_{0}^{2} }}\left[ {\frac{1}{{\left( {R^{4} + {\text{z}}_{0}^{2} R^{2} } \right)^{{{\raise0.7ex\hbox{$1$} \!\mathord{\left/ {\vphantom {1 2}}\right.\kern-\nulldelimiterspace} \!\lower0.7ex\hbox{$2$}}}} }} - \frac{1}{{R^{2} }}} \right]} \right\} \hfill \\ = \frac{1}{{{\text{z}}_{0}^{3} R^{3} }}\left\{ {\left( {R^{4} + {\text{z}}_{0}^{2} R^{2} } \right)^{{{\raise0.7ex\hbox{$1$} \!\mathord{\left/ {\vphantom {1 2}}\right.\kern-\nulldelimiterspace} \!\lower0.7ex\hbox{$2$}}}} - 2R^{2} + \frac{{R^{4} }}{{\left( {R^{4} + {\text{z}}_{0}^{2} R^{2} } \right)^{{{\raise0.7ex\hbox{$1$} \!\mathord{\left/ {\vphantom {1 2}}\right.\kern-\nulldelimiterspace} \!\lower0.7ex\hbox{$2$}}}} }}} \right\} \hfill \\ = \frac{1}{{{\text{z}}_{0}^{3} R^{3} }}\frac{{2R^{4} + {\text{z}}_{0}^{2} R^{2} - 2R^{2} \left( {R^{4} + {\text{z}}_{0}^{2} R^{2} } \right)^{{{\raise0.7ex\hbox{$1$} \!\mathord{\left/ {\vphantom {1 2}}\right.\kern-\nulldelimiterspace} \!\lower0.7ex\hbox{$2$}}}} }}{{\left( {R^{4} + {\text{z}}_{0}^{2} R^{2} } \right)^{{{\raise0.7ex\hbox{$1$} \!\mathord{\left/ {\vphantom {1 2}}\right.\kern-\nulldelimiterspace} \!\lower0.7ex\hbox{$2$}}}} }} \hfill \\ = \frac{{2R^{2} + {\text{z}}_{0}^{2} - 2\left( {R^{4} + {\text{z}}_{0}^{2} R^{2} } \right)^{{{\raise0.7ex\hbox{$1$} \!\mathord{\left/ {\vphantom {1 2}}\right.\kern-\nulldelimiterspace} \!\lower0.7ex\hbox{$2$}}}} }}{{{\text{z}}_{0}^{3} R\left( {R^{4} + {\text{z}}_{0}^{2} R^{2} } \right)^{{{\raise0.7ex\hbox{$1$} \!\mathord{\left/ {\vphantom {1 2}}\right.\kern-\nulldelimiterspace} \!\lower0.7ex\hbox{$2$}}}} }} \hfill \\ \end{gathered}$$

and

41 $$\begin{gathered} {\text{I}}_{3} = \frac{{z_{0} }}{2}\int\limits_{0}^{R} {\frac{{r^{2} }}{{R^{5} r_{{pi}} \left( {r_{{pi}} + 1} \right)}}} {\text{dr}}^{2} \hfill \\ = \frac{{z_{0} }}{{2R}}\int\limits_{0}^{R} {\frac{{r^{2} }}{{\left( {R^{4} + \left( {z_{0} r} \right)^{2} } \right)^{{{\raise0.7ex\hbox{$1$} \!\mathord{\left/ {\vphantom {1 2}}\right.\kern-\nulldelimiterspace} \!\lower0.7ex\hbox{$2$}}}} \left[ {\left( {R^{4} + \left( {z_{0} r} \right)^{2} } \right)^{{{\raise0.7ex\hbox{$1$} \!\mathord{\left/ {\vphantom {1 2}}\right.\kern-\nulldelimiterspace} \!\lower0.7ex\hbox{$2$}}}} + R^{2} } \right]}}} {\text{dr}}^{2} \hfill \\ = \frac{{z_{0} }}{{2R}}\frac{2}{{z_{0}^{2} }}\int\limits_{0}^{R} {\frac{{r^{2} }}{{\left( {R^{4} + \left( {z_{0} r} \right)^{2} } \right)^{{{\raise0.7ex\hbox{$1$} \!\mathord{\left/ {\vphantom {1 2}}\right.\kern-\nulldelimiterspace} \!\lower0.7ex\hbox{$2$}}}} + R^{2} }}} {\text{d}}\left( {R^{4} + \left( {z_{0} r} \right)^{2} } \right)^{{{\raise0.7ex\hbox{$1$} \!\mathord{\left/ {\vphantom {1 2}}\right.\kern-\nulldelimiterspace} \!\lower0.7ex\hbox{$2$}}}} \hfill \\ = \frac{{z_{0} }}{{2R}}\frac{2}{{z_{0}^{2} }}\int\limits_{0}^{R} {\frac{1}{{z_{0}^{2} }}\left[ {\frac{{R^{4} + \left( {z_{0} r} \right)^{2} }}{{\left( {R^{4} + \left( {z_{0} r} \right)^{2} } \right)^{{{\raise0.7ex\hbox{$1$} \!\mathord{\left/ {\vphantom {1 2}}\right.\kern-\nulldelimiterspace} \!\lower0.7ex\hbox{$2$}}}} + R^{2} }} - \frac{{R^{4} }}{{\left( {R^{4} + \left( {z_{0} r} \right)^{2} } \right)^{{{\raise0.7ex\hbox{$1$} \!\mathord{\left/ {\vphantom {1 2}}\right.\kern-\nulldelimiterspace} \!\lower0.7ex\hbox{$2$}}}} + R^{2} }}} \right]} {\text{d}}\left( {R^{4} + \left( {z_{0} r} \right)^{2} } \right)^{{{\raise0.7ex\hbox{$1$} \!\mathord{\left/ {\vphantom {1 2}}\right.\kern-\nulldelimiterspace} \!\lower0.7ex\hbox{$2$}}}} \hfill \\ {\text{ = }}\frac{{z_{0} }}{{2R}}\frac{2}{{z_{0}^{2} }}\frac{1}{{z_{0}^{2} }}\left[ {F_{1} + F_{2} } \right] \hfill \\ \end{gathered}$$

where

42 $${{F}_{1}}=\int\limits_{0}^{R}{\frac{{{R}^{4}}+{{\left( z_{0}^{{}}r \right)}^{2}}}{{{\left( {{R}^{4}}+{{\left( {{z}_{0}}r \right)}^{2}} \right)}^{{}^{1}\!\!\diagup\!\!{}_{2}\;}}+{{R}^{2}}}}\text{d}{{\left( {{R}^{4}}+{{\left( {{z}_{0}}r \right)}^{2}} \right)}^{{}^{1}\!\!\diagup\!\!{}_{2}\;}}$$

Take $y={{\left( {{R}^{4}}+{{\left( {{z}_{0}}r \right)}^{2}} \right)}^{{}^{1}\!\!\diagup\!\!{}_{2}\;}}$, then ${{y}_{0}}={{R}^{2}}$ and ${{y}_{R}}={{\left( {{R}^{4}}+{{\left( {{z}_{0}}\text{R} \right)}^{2}} \right)}^{1/2}}$ for r = 0 and r = R, respectively. Then we have

43 $$\begin{gathered} F_{1} = \int\limits_{{y_{0} }}^{{y_{R} }} {\frac{{y^{2} }}{{y + R^{2} }}} {\text{dy = }}\int\limits_{{y_{0} }}^{{y_{R} }} {\frac{{y^{2} - R^{4} + R^{4} }}{{y + R^{2} }}} {\text{dy = }}\int\limits_{{y_{0} }}^{{y_{R} }} {\left[ {\frac{{\left( {y{\text{ + R}}^{2} } \right)\left( {y - {\text{R}}^{2} } \right)}}{{y + R^{2} }}{\text{ + }}\frac{{R^{4} }}{{y + R^{2} }}} \right]} {\text{dy}} \hfill \\ {\text{ = }}\int\limits_{{y_{0} }}^{{y_{R} }} {\left[ {y - {\text{R}}^{2} {\text{ + }}\frac{{R^{4} }}{{y + R^{2} }}} \right]} {\text{dy = }}\left[ {\frac{1}{2}y^{2} - {\text{R}}^{2} {\text{y + }}R^{4} \ln \left| {y + R^{2} } \right|} \right]_{{y_{0} }}^{{y_{R} }} \hfill \\ = \frac{1}{2}\left( {Rz_{0} } \right)^{2} - R^{2} \left[ {\left( {R^{4} + \left( {Rz_{0} } \right)^{2} } \right)^{{1/2}} - R^{2} } \right] + R^{4} \left[ {\ln \left| {\left( {R^{4} + \left( {Rz_{0} } \right)^{2} } \right)^{{1/2}} + R^{2} } \right| - \ln \left( {2R^{2} } \right)} \right] \hfill \\ \end{gathered}$$

And

44 $$\begin{gathered} F_{2} = - \int\limits_{0}^{R} {\frac{{R^{4} }}{{\left( {R^{4} + \left( {z_{0} r} \right)^{2} } \right)^{{{\raise0.7ex\hbox{$1$} \!\mathord{\left/ {\vphantom {1 2}}\right.\kern-\nulldelimiterspace} \!\lower0.7ex\hbox{$2$}}}} + R^{2} }}} {\text{d}}\left( {R^{4} + \left( {z_{0} r} \right)^{2} } \right)^{{{\raise0.7ex\hbox{$1$} \!\mathord{\left/ {\vphantom {1 2}}\right.\kern-\nulldelimiterspace} \!\lower0.7ex\hbox{$2$}}}} \hfill \\ - \int\limits_{{y_{0} }}^{{y_{R} }} {\frac{{R^{4} }}{{y + R^{2} }}} {\text{dy = }} - R^{4} \left[ {\ln \left| {\left( {R^{4} + \left( {Rz_{0} } \right)^{2} } \right)^{{1/2}} + R^{2} } \right| - \ln \left( {2R^{2} } \right)} \right] \hfill \\ \end{gathered}$$

Take Eqs. (43, 44) into Eq. (41), we have

45 $$\begin{gathered} {\text{I}}_{3} = \frac{{z_{0} }}{{2R}}\frac{2}{{z_{0}^{2} }}\frac{1}{{z_{0}^{2} }}\left[ {F_{1} + F_{2} } \right] \hfill \\ {\text{ = }}\frac{{z_{0} }}{{2R}}\frac{2}{{z_{0}^{2} }}\frac{1}{{z_{0}^{2} }}\frac{1}{2}\left\{ {\left( {Rz_{0} } \right)^{2} -2 R^{2} \left[ {\left( {R^{4} + \left( {Rz_{0} } \right)^{2} } \right)^{{1/2}} - R^{2} } \right]} \right\} \hfill \\ {\text{ = }}\frac{1}{{2Rz_{0}^{3} }}\left\{ {\left( {Rz_{0} } \right)^{2} - 2R^{2} \left[ {\left( {R^{4} + \left( {Rz_{0} } \right)^{2} } \right)^{{1/2}} - R^{2} } \right]} \right\} \hfill \\ \hfill \\ {\text{ = }}\frac{1}{{2Rz_{0}^{3} }}\left[ {\left( {Rz_{0} } \right)^{2} - 2R^{2} \left( {R^{4} + \left( {Rz_{0} } \right)^{2} } \right)^{{1/2}} + 2R^{4} } \right] \hfill \\ \end{gathered}$$

Finally, we have

46 $$\begin{gathered} {\text{I}} = - \frac{{P_{{\text{z}}} }}{\sigma }\left[ {I_{1} + I_{2} + I_{3} } \right]{\text{ = }} - \frac{{P_{{\text{z}}} }}{\sigma }\left\{ { - \frac{{z_{0} }}{{\left( {{\text{R}}^{2} + {\text{z}}_{0}^{2} } \right)^{{{\raise0.7ex\hbox{$1$} \!\mathord{\left/ {\vphantom {1 2}}\right.\kern-\nulldelimiterspace} \!\lower0.7ex\hbox{$2$}}}} }} + 1{\text{ + }}\frac{{2R^{2} + {\text{z}}_{0}^{2} - 2\left( {R^{4} + {\text{z}}_{0}^{2} R^{2} } \right)^{{{\raise0.7ex\hbox{$1$} \!\mathord{\left/ {\vphantom {1 2}}\right.\kern-\nulldelimiterspace} \!\lower0.7ex\hbox{$2$}}}} }}{{{\text{z}}_{0}^{3} R\left( {R^{4} + {\text{z}}_{0}^{2} R^{2} } \right)^{{{\raise0.7ex\hbox{$1$} \!\mathord{\left/ {\vphantom {1 2}}\right.\kern-\nulldelimiterspace} \!\lower0.7ex\hbox{$2$}}}} }}} \right. \hfill \\ \left. {{\text{ + }}\frac{1}{{2Rz_{0}^{3} }}\left[ {\left( {Rz_{0} } \right)^{2} - 2R^{2} \left( {R^{4} + \left( {Rz_{0} } \right)^{2} } \right)^{{1/2}} + 2R^{4} } \right]} \right\} \hfill \\ {\text{ = }} - \frac{{P_{{\text{z}}} }}{\sigma }\left\{ { - \frac{{z_{0} }}{{\left( {{\text{R}}^{2} + {\text{z}}_{0}^{2} } \right)^{{{\raise0.7ex\hbox{$1$} \!\mathord{\left/ {\vphantom {1 2}}\right.\kern-\nulldelimiterspace} \!\lower0.7ex\hbox{$2$}}}} }} + 1{\text{ + }}\frac{{2R^{2} + {\text{z}}_{0}^{2} - 2\left( {R^{4} + {\text{z}}_{0}^{2} R^{2} } \right)^{{{\raise0.7ex\hbox{$1$} \!\mathord{\left/ {\vphantom {1 2}}\right.\kern-\nulldelimiterspace} \!\lower0.7ex\hbox{$2$}}}} }}{{{\text{z}}_{0}^{3} R^{2} \left( {R^{2} + {\text{z}}_{0}^{2} } \right)^{{{\raise0.7ex\hbox{$1$} \!\mathord{\left/ {\vphantom {1 2}}\right.\kern-\nulldelimiterspace} \!\lower0.7ex\hbox{$2$}}}} }}} \right. \hfill \\ \left. {{\text{ + }}\frac{1}{{2z_{0}^{3} R^{2} }}\left[ {{\text{R}}\left( {Rz_{0} } \right)^{2} - 2R^{3} \left( {R^{4} + \left( {Rz_{0} } \right)^{2} } \right)^{{1/2}} + 2R^{5} } \right]} \right\} \hfill \\ {\text{ = }} - \frac{{P_{{\text{z}}} }}{\sigma }\frac{{ - 2{\text{z}}_{0}^{4} R^{2} + 2{\text{z}}_{0}^{3} R^{2} \left( {R^{2} + {\text{z}}_{0}^{2} } \right)^{{{\raise0.7ex\hbox{$1$} \!\mathord{\left/ {\vphantom {1 2}}\right.\kern-\nulldelimiterspace} \!\lower0.7ex\hbox{$2$}}}} + 4R^{2} + 2{\text{z}}_{0}^{2} - 4\left( {R^{4} + {\text{z}}_{0}^{2} R^{2} } \right)^{{{\raise0.7ex\hbox{$1$} \!\mathord{\left/ {\vphantom {1 2}}\right.\kern-\nulldelimiterspace} \!\lower0.7ex\hbox{$2$}}}} + {\text{R}}\left( {Rz_{0} } \right)^{2} \left( {R^{2} + {\text{z}}_{0}^{2} } \right)^{{{\raise0.7ex\hbox{$1$} \!\mathord{\left/ {\vphantom {1 2}}\right.\kern-\nulldelimiterspace} \!\lower0.7ex\hbox{$2$}}}} - 2R^{4} \left( {R^{2} + {\text{z}}_{0}^{2} } \right) + 2R^{5} \left( {R^{2} + {\text{z}}_{0}^{2} } \right)^{{{\raise0.7ex\hbox{$1$} \!\mathord{\left/ {\vphantom {1 2}}\right.\kern-\nulldelimiterspace} \!\lower0.7ex\hbox{$2$}}}} }}{{2{\text{z}}_{0}^{3} R^{2} \left( {R^{2} + {\text{z}}_{0}^{2} } \right)^{{{\raise0.7ex\hbox{$1$} \!\mathord{\left/ {\vphantom {1 2}}\right.\kern-\nulldelimiterspace} \!\lower0.7ex\hbox{$2$}}}} }} \hfill \\ = - \frac{{P_{{\text{z}}} }}{\sigma }\frac{{ - 2{\text{z}}_{0}^{4} R^{2} + 2{\text{z}}_{0}^{3} R^{2} \left( {R^{2} + {\text{z}}_{0}^{2} } \right)^{{{\raise0.7ex\hbox{$1$} \!\mathord{\left/ {\vphantom {1 2}}\right.\kern-\nulldelimiterspace} \!\lower0.7ex\hbox{$2$}}}} + 4R^{2} + 2{\text{z}}_{0}^{2} - 4R\left( {R^{2} + {\text{z}}_{0}^{2} } \right)^{{{\raise0.7ex\hbox{$1$} \!\mathord{\left/ {\vphantom {1 2}}\right.\kern-\nulldelimiterspace} \!\lower0.7ex\hbox{$2$}}}} + R^{3} z_{0}^{2} \left( {R^{2} + {\text{z}}_{0}^{2} } \right)^{{{\raise0.7ex\hbox{$1$} \!\mathord{\left/ {\vphantom {1 2}}\right.\kern-\nulldelimiterspace} \!\lower0.7ex\hbox{$2$}}}} - 2R^{6} - 2R^{4} z_{0}^{2} + 2R^{5} \left( {R^{2} + {\text{z}}_{0}^{2} } \right)^{{{\raise0.7ex\hbox{$1$} \!\mathord{\left/ {\vphantom {1 2}}\right.\kern-\nulldelimiterspace} \!\lower0.7ex\hbox{$2$}}}} }}{{{\text{2z}}_{0}^{3} R^{2} \left( {R^{2} + {\text{z}}_{0}^{2} } \right)^{{{\raise0.7ex\hbox{$1$} \!\mathord{\left/ {\vphantom {1 2}}\right.\kern-\nulldelimiterspace} \!\lower0.7ex\hbox{$2$}}}} }} \hfill \\ = - \frac{{P_{{\text{z}}} }}{\sigma }\frac{{ - 2{\text{z}}_{0}^{4} R^{2} + \left( { - 4R + 2{\text{z}}_{0}^{3} R^{2} + z_{0}^{2} R^{3} + 2R^{5} } \right)\left( {R^{2} + {\text{z}}_{0}^{2} } \right)^{{{\raise0.7ex\hbox{$1$} \!\mathord{\left/ {\vphantom {1 2}}\right.\kern-\nulldelimiterspace} \!\lower0.7ex\hbox{$2$}}}} + 2{\text{z}}_{0}^{2} + 4R^{2} - 2z_{0}^{2} R^{4} - 2R^{6} }}{{{\text{2z}}_{0}^{3} R^{2} \left( {R^{2} + {\text{z}}_{0}^{2} } \right)^{{{\raise0.7ex\hbox{$1$} \!\mathord{\left/ {\vphantom {1 2}}\right.\kern-\nulldelimiterspace} \!\lower0.7ex\hbox{$2$}}}} }} \hfill \\ \end{gathered}$$

Specifically, let $\sigma {\text{ = }}1$, ${\text{P}}_{z} {\text{ = 1}}$, *R* = 1, we have

47 $$\begin{gathered} {\text{I}} = - \frac{{ - 2{\text{z}}_{0}^{4} + \left( { - 4 + 2{\text{z}}_{0}^{3} + z_{0}^{2} + 2} \right)\left( {1 + {\text{z}}_{0}^{2} } \right)^{{{\raise0.7ex\hbox{$1$} \!\mathord{\left/ {\vphantom {1 2}}\right.\kern-\nulldelimiterspace} \!\lower0.7ex\hbox{$2$}}}} + 2{\text{z}}_{0}^{2} + 4 - 2z_{0}^{2} - 2}}{{{\text{2z}}_{0}^{3} \left( {1 + {\text{z}}_{0}^{2} } \right)^{{{\raise0.7ex\hbox{$1$} \!\mathord{\left/ {\vphantom {1 2}}\right.\kern-\nulldelimiterspace} \!\lower0.7ex\hbox{$2$}}}} }} \hfill \\ {\text{ = }} - \frac{{ - 2{\text{z}}_{0}^{4}{\text{ + }}\left( { - 2 + 2{\text{z}}_{0}^{3} + z_{0}^{2} } \right)\left( {1 + {\text{z}}_{0}^{2} } \right)^{{{\raise0.7ex\hbox{$1$} \!\mathord{\left/ {\vphantom {1 2}}\right.\kern-\nulldelimiterspace} \!\lower0.7ex\hbox{$2$}}}} + 2}}{{{\text{2z}}_{0}^{3} \left( {1 + {\text{z}}_{0}^{2} } \right)^{{{\raise0.7ex\hbox{$1$} \!\mathord{\left/ {\vphantom {1 2}}\right.\kern-\nulldelimiterspace} \!\lower0.7ex\hbox{$2$}}}} }} \hfill \\ \end{gathered}$$

For Eq. (47), it is easy to test by Matlab that its value is always smaller than 0 and $z_{0}$ value dependent. For example, for $z_{0}$ = 0.001, 0.01, 0.05, 0.1, 0.2, 0.3, 0.4, 0.5, 0.6, 0.7, 0.8, 0.9, 0.99, the values of I are −0.9994, −0.9938, −0.9688, −0.9377, −0.8765, −0.8173, −0.7611, −0.7082, −0.6591, −0.6138, −0.5723, −0.5343, -0.5033, respectively. In fact, here the dipole is oriented along the positive z-axis in the upper semi-sphere, the potential on the circualr plane would be negative.

Specifically, if *R* tends to infinity, then according to Eq. (38), we have

48 $$\text{I}=\int\limits_{0}^{2\pi }{\int\limits_{0}^{R}{\Phi }rd\theta }dr=-\frac{{{P}_{\text{z}}}}{\sigma }\int\limits_{0}^{R}{\left[ \frac{{{z}_{0}}}{r_{p}^{3}}+\frac{{{r}^{2}}{{z}_{0}}}{{{R}^{5}}r_{pi}^{3}}\text{+}\frac{{{r}^{2}}{{z}_{0}}}{{{R}^{5}}{{r}_{pi}}\left( {{r}_{pi}}+1 \right)} \right]}r\text{dr}=-\frac{{{P}_{\text{z}}}}{\sigma }{{I}_{1}}\text{=}-\frac{{{P}_{\text{z}}}}{\sigma }$$

It is the same as Eq. (10) except a sign because the dipole here is located upper the plane, and it is below the plane for Eq. (10).
